# Variants in *BMP7* and *BMP15* 3’-*UTR*s Associated with Reproductive Traits in a Large White Pig Population

**DOI:** 10.3390/ani9110905

**Published:** 2019-11-01

**Authors:** Hang Yin, Xing Du, Qiqi Li, Zengxiang Pan, Wangjun Wu, Honglin Liu, Qifa Li

**Affiliations:** College of Animal Science and Technology, Nanjing Agricultural University, Nanjing 210095, China; 18151665587@163.com (H.Y.); duxing@njau.edu.cn (X.D.); 2015105012@njau.edu.cn (Q.L.); owwa@njau.edu.cn (Z.P.); wuwangjun2012@njau.edu.cn (W.W.); liuhonglin@njau.edu.cn (H.L.)

**Keywords:** *BMP7*, *BMP15*, reproductive trait, variant, Large White pig

## Abstract

Bone morphogenetic protein 7 (*BMP7*) and *BMP15*, which encode members of the BMP family, have been identified by whole-genome resequencing as breeding-related genes that overlap with a known quantitative trait locus for reproductive traits. In this study, we investigated the effects of variants at the *BMP7* and *BMP15* gene loci on sow reproductive traits. We isolated 669 and 1213 bp sequences of the 3’-untranslated region (3’-*UTR*) of the porcine *BMP7* and *BMP15* genes, respectively, and detected several RNA regulatory elements, such as miRNA response elements and AU-rich elements. Pooled DNA sequencing identified two novel point mutations (viz., *BMP7* c.1569A>G and *BMP15* c.2366G>A) in the 3’-*UTR*. Association analysis showed that the c.1569A>G polymorphism was associated with the litter weight trait in a Large White pig population. Furthermore, analysis of the combined genetic effects revealed that *AA*/*GA* and *AG*/*GG* were the favorable combined genotypes for the total number of piglets born (TNB) and the total number of piglets born alive (NBA), whereas. Together, our findings confirm that *BMP7* and *BMP15* are candidate genes for porcine reproductive performance.

## 1. Introduction

Bone morphogenetic proteins (BMPs) are a group of multifunctional cytokines that belong to the BMP subfamily of the transforming growth factor beta (TGF-β) superfamily. As secreted signaling molecules and ligands, BMPs often exert their biological functions (e.g., ovarian functions) by forming the BMP/SMAD signaling pathway with receptors such as bone morphogenetic protein receptor type 1A (BMPR1A), BMPR1B, and BMPR2, and SMAD proteins (including SMAD1, SMAD5, SMAD8, and SMAD4) [1,2]. In the ovary, BMPs first interact with their heterotetrameric receptor complexes on the surface of granulosa cells (GCs). As an oocyte-secreted growth factor, *BMP15* usually forms a homodimer or heterodimer with another oocyte-secreted growth factor, growth differentiation factor 9 (*GDF9*), before interacting with its receptors [3]. In the cytoplasm, these receptors activate SMAD1, SMAD5, and SMAD8 by mediating their phosphorylation, and the activated SMADs then form complexes with SMAD4. These SMAD complexes subsequently enter the nucleus where they control several key genes for follicular development, oocyte maturation and ovulation, and luteal formation by acting as a transcription factor [4,5,6]. 

In mammals, the BMPs are known to be essential for female fertility. Increasing evidence suggests that the dysregulation and dysfunction of the BMPs can cause follicular development arrest, ovulation disorders, decreased ovulation rate and litter size, and even infertility and other ovarian diseases [7,8]. Notably, in domestic animals, the BMPs are either major genes for high fecundity or candidate genes for reproductive traits [9,10,11,12,13]. In sheep, *BMP15* and *BMPR1B* are the major genes for high fecundity traits, where the *FecB* mutation of *BMPR1B,* in particular, has been widely used in sheep breeding [9,10,11]. In Large White and Taihu sows, *BMP7* and *BMP15* have been identified through whole-genome resequencing to be breeding-related genes that overlap with a known quantitative trait locus for reproductive traits, respectively [12,13]. However, variants that affect reproductive performance have not been found in the 3’-untranslated region (3’-*UTR*) of the porcine *BMP7* and *BMP15* genes. Therefore, we aimed to characterize the 3’-*UTR* of these two porcine genes in this study. We also aimed to screen mutations in these regions, and to understand the relationship between the mutations and reproductive performance in a Large White pig population.

## 2. Materials and Methods

### 2.1. Samples

Ear samples of Large White sows (*n* = 227) were randomly collected from Jiangsu Kangle Farming Co. (Changzhou, China). Their reproductive traits are listed in Supplementary Table S1. All animal-related experiments were approved by the Animal Ethics Committee at Nanjing Agricultural University, China (SYXK 2017-0027).

### 2.2. Genomic DNA Extraction

DNA was extracted from the ear samples using the conventional phenol–chloroform method. In brief, the ear tissues from Large White sows were lyzed by DNA lysis buffer and proteinase K for 12 h and the impurities were separated by using Tris saturated phenol, chloroform or isoamyl alcohol in sequence. Finally, the genomic DNA was extracted and stored at −20 °C for further analysis. 

### 2.3. Primer Design 

The specific primers used to isolate the 3’-*UTR* sequences of the Large White pig *BMP7* and *BMP15* genes were designed using Primer Premier v5.0 software (PREMIER Biosoft, Palo Alto, CA, USA), according to the sequences of the porcine *BMP7* and *BMP15* genes provided by the NCBI database (https://www.ncbi.nlm.nih.gov/). The primers (listed in Table 1) were synthesized by Tsingke Biological Technology (Tsingke, Beijing, China).

### 2.4. PCR Amplification and Sequencing

Polymerase chain reactions (PCRs) were performed in a 20 μL volume containing 10 μL of 2× VazymeLAmp^®^ Master Mix, 7 μL of ddH_2_O, 1 μL of forward primer, 1 μL of reverse primer, and 1 μL of DNA. The PCR cycles were as follows: 5 min at 94 °C, then 35 cycles of 30 s at 94 °C, 30 s at the annealing temperature (Table 1), 1 min at 72 °C, and finally, 7 min at 72 °C. PCR products were identified by 1.5% agarose gel electrophoresis and only the single and clear bands that fit for the expected length were selected for sanger sequencing (Tsingke, Beijing, China).

### 2.5. Sequence Analysis

DNAStar v5.22 software (DNASTAR, Madison, WI, USA) was used to analyze the nucleotide sequences of the 3’-*UTR*. The miRBase and miRTarBase websites were used to predict the microRNA (miRNA) response elements (MREs) within the *BMP7* and *BMP15* 3’-*UTR* sequences.

### 2.6. Genotyping

The point mutations in the 3’-*UTR* of the porcine *BMP7* and *BMP15* genes were genotyped by direct sequencing; the specific primers used are listed in Table 1.

### 2.7. Association Analysis

The general linear model of the SAS v9.2 software package (SAS Institute Inc., Cary, NC, USA) was applied for analysis of the effects of different parities and genotypes on various reproductive traits of Large White pigs; namely, the total number of piglets born (TNB), the total number of piglets born alive (NBA), number of stillborns (NSB), and litter weight (LW). The statistical model was y_iklmn_ = µ + HYS_i_ + P_k_ + A_l_ + G_m_ + e_iklmn_, where y_iklmn_ is the individual observation for the traits, μ is the overall population mean, HYSi is the effect of hoggery-year-season, P_k_ is the effect of parity, A_l_ is the effect of age (days), G_m_ is the effect of the gene, and e_iklmn_ is the random residual effect.

## 3. Results

### 3.1. Isolation and Characterization of the 3’-UTR of the Porcine BMP7 Gene 

**Figure 1:**
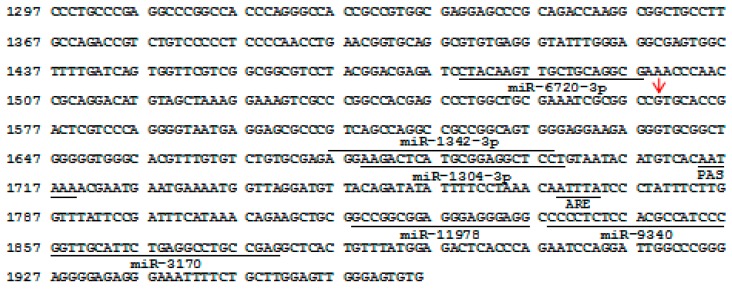
Characterization of the partial sequence of the 3’-untranslated region of the Large White pig bone morphogenetic protein 7 (*BMP7*) gene. The transcription start codon was assumed as +1 (GenBank ID: XM_005673044.3). The underline indicates the regulatory elements. Red arrows indicate the mutation c.1569A>G.

The partial sequence of the 3’-*UTR* of *BMP7* was isolated by PCR amplification and sequencing (Figure 1). Several classic regulatory elements, such as the poly(A) signal (PAS; AAUAAA) and AU-rich element (ARE; AUUUA), were identified within this region; that is, at c.1714/1719, and c.1769/1773 (the transcription start codon of the porcine *BMP7* was assumed as +1). In addition, the putative response elements for *miR-6720-3p*, *miR-1342-3p*, *miR-1304-3p*, *miR-11978*, *miR-9340*, and *miR-3170* were predicted at c.1479/1497, c.1676/1698, c.1679/1699, c.1818/1836, c.1838/1858, and c.1859/1880, respectively.

### 3.2. Polymorphism of the 3’-UTR of the Porcine BMP7 Gene 

**Figure 2:**
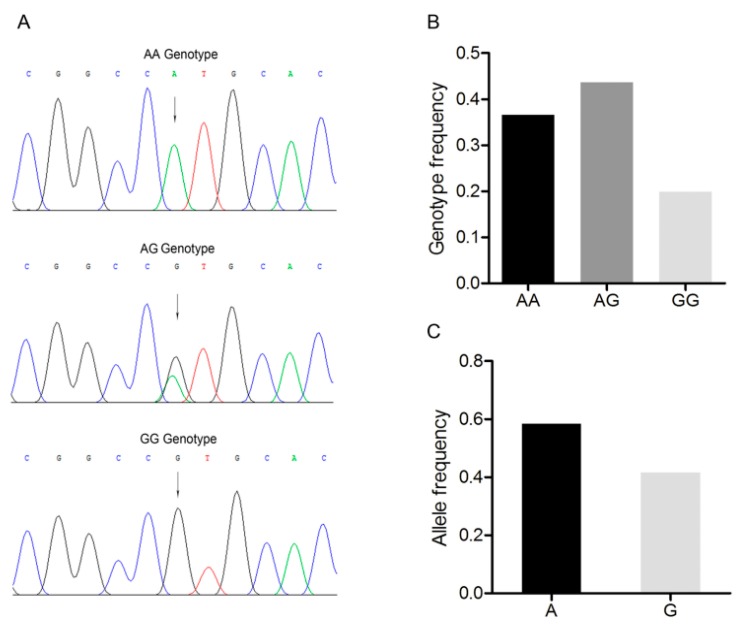
Mutation c.1569A>G in the 3’-untranslated region of the Large White pig *BMP7* gene. (**A**) Sequence of different genotypes at the mutation c.1569A>G. The arrow indicates the substitution position. (**B**) Genotype frequency of the mutation c.1569A>G. (**C**) Allele frequency of the mutation c.1569A>G. *n* = 227.

An A/G point mutation site was detected in this region and designated as c.1569A>G (Figure 2A). Three genotypes, *AA*, *AG*, and *GG* were discovered in the Large White pig population (Figure 2A,B). *A* was the dominant allele with a frequency of 0.600, and *AG* was the dominant genotype with a frequency of 0.449 (Figure 2C). The Chi-square test indicated that this single nucleotide polymorphism fitted the Hardy–Weinberg equilibrium (*p* > 0.05).

### 3.3. Association Analysis between the BMP7 c.1569A>G Polymorphism and Reproductive Traits

The effects of the point mutation c.1569A>G on the TNB, NBA, NSB, and LW traits of Large White pigs were determined using a mixed model. The results showed that the LW of sows with the *AG* genotype was significantly higher than that of sows with genotype *GG* (*p* < 0.05) or *AA* (*p* < 0.01) (Table 2, and Supplementary Table S2). Although the effects of *AA* genotype and *GG* genotype on TNB and NBA have no significant difference in statistics, it is worth noting that the TNB and NBA of sows with the *AA* genotype are 0.61 and 0.47 per parity are higher than that of sows with the *GG* genotype, respectively (Table 2). 

### 3.4. Isolation and Characterization of the 3’-UTR of the Porcine BMP15 Gene 

**Figure 3:**
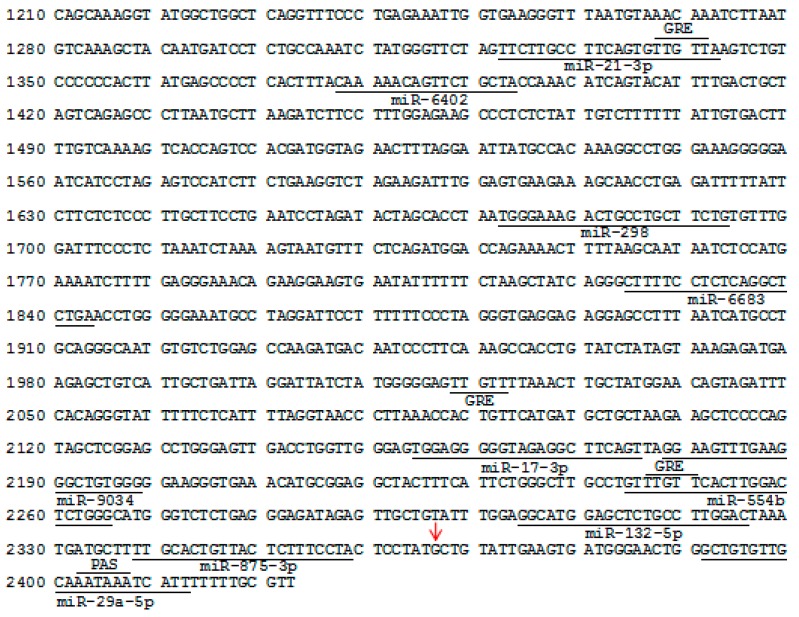
Characterization of the partial sequence of the 3’-untranslated region of the Large White pig *BMP15* gene. The transcription start codon was assumed as +1 (GenBank ID: NM_001005155.2). The underline indicates the regulatory elements. Red arrows indicate the mutation c.2366G>A.

We next isolated a 1213-bp sequence of the 3’-*UTR* of *BMP15* gene from the Large White pig (Figure 3) and found that this was also a gene containing a PAS motif. The partial sequence contained three classic GU-rich element (GRE; UUGUU) motifs located at c.1337/1341 (GRE1), c.2018/2022 (GRE2), and c.2246/2250 (GRE3), and a PAS motif located at c.2402/2407 (the transcription start codon of the porcine *BMP15* was assumed as +1), but ARE motifs were not detected. Furthermore, ten target sites of miRNAs were detected in the partial 3’-*UTR* sequence, such as *miR-21-3p* (c.1322/1342), *miR-298* (c.1672/1693), *miR-17-3p* (c.2154/2175), *miR-132-5p* (c.2204/2225), and *miR-29a-5p* (c.2391/2412).

### 3.5. Detection of a G/A Point Mutation Site in the 3’-UTR of the BMP15 Gene

**Figure 4:**
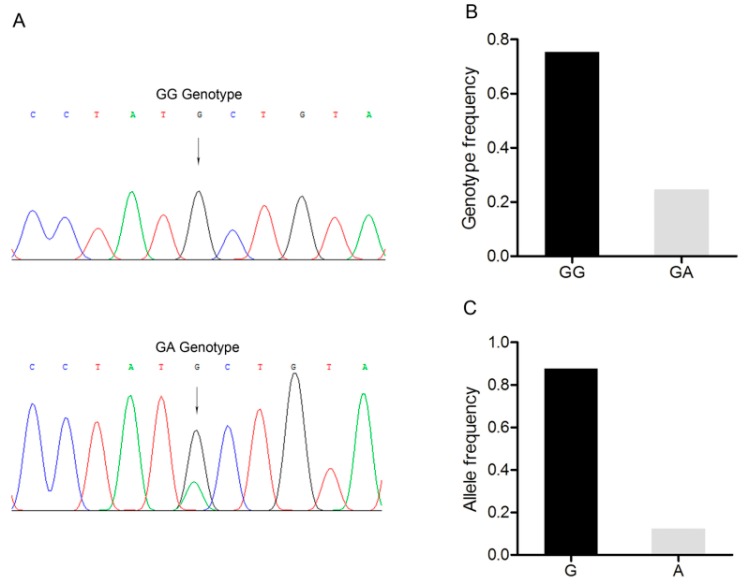
Mutation c.2366G>A in the 3’-untranslated region of the Large White pig *BMP15* gene. (**A**) Sequence of different genotypes at the mutation c.2366G>A. The arrow indicates the substitution position. (**B**) Genotype frequency of the mutation c.2366G>A. (**C**) Allele frequency of the mutation c.2366G>A. *n* = 227.

A G/A point mutation site was identified in the 3’-*UTR* of the *BMP15* gene and designated as c.2366G>A (Figure 4A). Only two genotypes, *GG* and *GA*, were discovered in the Large White pig population (*n* = 227) (Figure 4B). *G* was the dominant allele with a frequency of 0.866, and *GG* was the dominant genotype with a frequency of 0.732 (Figure 4C). The Chi-square test showed that this point mutation fitted the Hardy–Weinberg equilibrium (*p* > 0.05).

### 3.6. Association Analysis between the BMP15 c.2366G>A Polymorphism and Reproductive Traits

Analyses of the effect of the point mutation c.2366G>A on the various reproductive traits revealed that this polymorphism was not significantly effective on any of the traits in the Large White pig population (*p* > 0.05) (Table 3).

### 3.7. Association Analysis between the Combined Genotypes of BMP7 c.1569A>G and BMP15 c.2366G>A and Reproductive Traits

**Figure 5:**
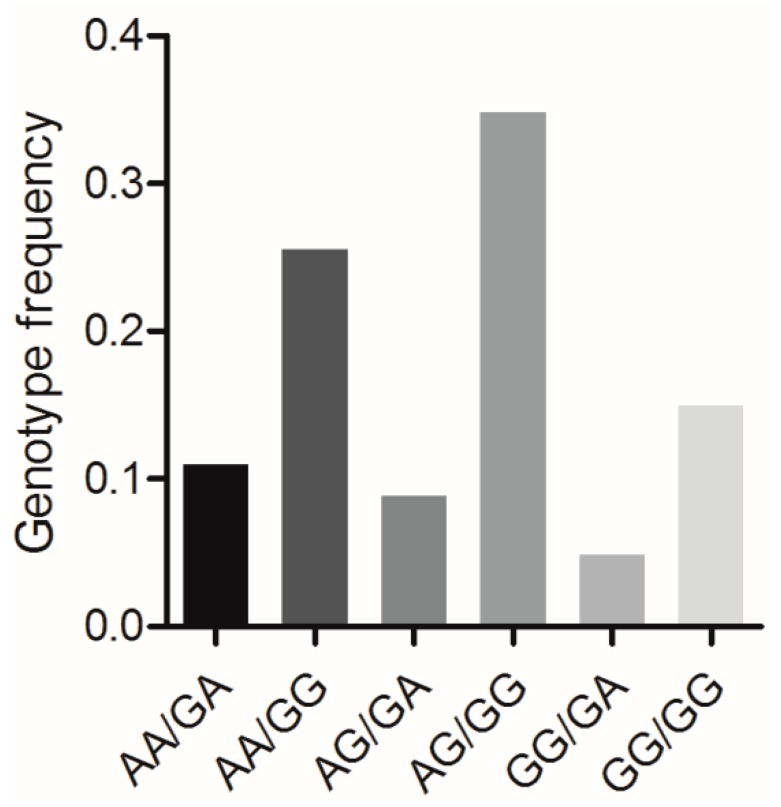
Genotype frequencies of the mutations *BMP7* c.1569A>G and *BMP15* c.2366G>A in Large White pig population. *n* = 227.

*BMP7* c.1569A>G and *BMP15* c.2366G>A formed six combined genotypes (viz., *AA*/*GA*, *AA*/*GG*, *AG*/*AG*, *AG*/*GG*, *GG*/*GA*, and *GG*/*GG*) in the Large White pig population (*n* = 227) (Figure 5). Of these, the frequency of genotype *AG*/*GG* was the highest (0.348), whereas that of genotype *GG*/*GA* was the lowest (0.048). The effects of the combined genotypes on the various reproductive traits in this pig population are shown in Table 4. For the TNB and NBA traits, sows with the *AA*/*GA* and *AG/GG* genotypes had significantly higher numbers than sows with the genotype *AG/GA* (*p* < 0.05). 

## 4. Discussion

The 3’-*UTR*s are the multi-functional components of mRNAs, representing a central regulatory hub that recruits RNA-binding proteins (RBPs) and non-coding RNAs (ncRNAs) to control mRNA translation, localization, stability, and the polyadenylation status [14,15]. Recognition sites for RBPs and ncRNAs (also known as RNA regulatory elements) have been shown to mediate the 3’-*UTR-*determined gene expression level in various tissues and cells [15,16]. In this study, we isolated and characterized partial sequences of the 3’-*UTR*s of the porcine *BMP7* and *BMP15* genes, and multiple RNA regulatory elements such as miRNA response elements (MREs), GREs, and AREs were detected. MREs are sequences in the 3’-*UTR* of mRNAs that recognize the seed region on the miRNA, thereby mediating direct interactions between the miRNA and its target mRNA [16,17]. Importantly, several putative *BMP7*- or *BMP15*-targeted miRNAs, such as *miR-17-3p* [18], *miR-21-3p* [19], *miR-29a-5p* [20], and *miR-132-5p* [21], have been demonstrated to be related to reproduction [22,23]. The ARE motif, an important *cis*-element for RNA regulation, is involved in RNA processing, transport, and translation through its interaction with ARE-binding proteins, such as the tristetraprolin (TTP), heterogeneous nuclear ribonucleoprotein D (HNRPD; also AUF1), ELAV-like RNA-binding protein 1 (ELAV1; also HuR), and KH-type splicing regulatory protein (KSRPH) [24,25]. The GRE motif, a conserved sequence enriched in the 3’-*UTR* of mRNAs, which mediated regulation of GRE-binding proteins (e.g., nucleolin and fragile X mental retardation protein) on mRNA stability [26,27]. However, the regulation of porcine *BMP7* and *BMP15* genes by these RNA regulatory elements has not been experimentally verified and needs further investigation.

*BMP7* is an important ligand of the BMP/SMAD signaling pathway, which plays a critical role in steroidogenesis, follicular development, and female fertility [13,28]. In the mammalian ovary, *BMP7* is highly expressed in the GCs and theca cells (TCs) of dominant follicles [28]. In follicular cells cultured in vitro, the inhibition of *BMP7* induced the suppression of androgen secretion by bovine ovarian TCs [29], whereas the addition of recombinant *BMP7* stimulated estrogen (E2) release by buffalo ovarian GCs [28], as well as progesterone (P4) production by human ovarian granulosa lutein cells [30], and buffalo ovarian luteal cells [31]. *BMP7* also promoted GC survival in the buffalo ovary [28] and GC proliferation in the rat ovary [32]. Injections of *BMP7* into the ovarian bursa of rats increased the numbers of primordial, primary, preantral, and antral follicles, and decreased the ovulation rate and serum P4 levels [32]. Furthermore, both ovarian *BMP7* levels and *BMP7* polymorphisms have been shown to be associated with fertility in domestic animals [13,33]. In sheep, the *BMP7* mRNA levels were significantly higher in the ovarian follicles of *FecB*-carrying ewes with high fecundity [33]. High-throughput technology showed that *BMP7* was a candidate gene for reproductive traits in Large White pigs [13] and for the high prolificacy of Hu sheep [34]. In this study, we identified the novel point mutation c.1569A>G in the 3’-*UTR* of the porcine *BMP7* gene and found that its polymorphism was associated with the LW trait in a Large White pig population. In three pig populations (Landrace, Large White, and Duroc), the g.35161T>C polymorphism was shown to be significantly associated with the NBA and LW traits (*p* < 0.05), and the LW at 21 days (*p* < 0.01) [35]. Our findings further demonstrated that *BMP7* is a candidate gene for the reproductive traits of sow, and would be a novel genetic marker for marker-assisted selection in pig breeding. 

*BMP15* is an important cytokine that is expressed specifically in the ovaries of mammals [3]. As an oocyte-secreted growth factor, *BMP15* plays paracrine/autocrine roles in regulating the functions of GCs (e.g., proliferation, differentiation, and apoptosis) and in stimulating the action of follicle-stimulating hormone (FSH), the expansion of cumulus cells, and ovulation [36,37,38]. Importantly, *BMP15* has been shown to be essential for female fertility in most mammalian species; in particular, homozygosity for *BMP15* mutations leads to subfertility in mice and sterility in sheep [39,40]. In addition, the *BMP15* levels and *GDF9*:*BMP15* ratio have been demonstrated to be directly correlated with the litter size in mammals [36,41]. *BMP15* has been identified as a major gene for high fecundity traits, including the litter size and ovulation rate in sheep, and multiple mutations (e.g., *FecX*^B^, *FecX*^Bar^, *FecX*^G^, *FecX*^Gr^, *FecX*^H^, *FecX*^I^, *FecX*^L^, *FecX*^O^, and *FecX*^R^) have been proven to significantly improve fecundity [9,11,42]. Recently, the novel haplotype variant *FecX*^Bar^ that consists of three polymorphisms (viz., c.301G>T, c.302_304delCTA, and c.310insC) was observed in exon 1 of the *BMP15* gene of the W flock (a strain line originally created using prolific Barbarine ewes), and this allele increased the ovulation rate by 0.7 and the litter size by 0.3 lambs [43]. In Taihu pigs, *BMP15* has been identified through whole-genome resequencing to be a breeding-related gene that overlaps with a known quantitative trait locus for reproductive traits [12]. However, no variant has been found in the porcine *BMP15* gene until now. In this study, we identified the novel point mutation c.2366G>A in the 3’-*UTR* of this gene, but it had no significant effects on the reproductive traits in a Large White pig population. Thus, further screening for *BMP15* gene variants that affect sow reproductive traits is needed.

Reproductive traits are the complex economic traits controlled by the cumulative small effects of multiple genes [13,44]. In some cases, the selection of a single locus or a single gene is insufficient to improve the reproductive performance of sows in the pig breeding industry. Therefore, the analysis of the combined genetic effects of multiple genes or multiple loci on reproductive traits has attracted increasing attention [45,46]. For instance, in Hirschmann hybrid-line sows, two point mutations (657C>T and 749G>C) in intron 6 of the porcine pregnancy-associated glycoprotein 2-like subfamily (*pPAG2-Ls*) gene were combined into a diplotype; subsequently, significant associations of the NBA trait with the combined genotype 657CC/749GC (12.71 ± 0.47) were shown compared with 657CT/749GG (11.39 ± 0.22) [45]. A recent report described 26 putative lethal haplotypes in a Finnish Yorkshire population (observed using genome-wide association analysis), where haplotype 8-2026 on chromosome 8 (position 107.0–113.3 Mb, 135 marker) was significantly associated with the NSP trait [47]. In this study, we showed that *BMP7* c.1569A>G and *BMP15* c.2366G>A formed six combined genotypes, of which *AA*/*GA* and *AG/GG* were preferable for sows, being associated with higher TNB and NBA. Similarly, six combined genotypes that were favorable for reproductive traits (viz., estrogen receptor (*ESR*)*^AA^*/*FSHb^BB^*, *ESR^AA^*/catenin alpha-like 1 (*CTNNAL1*)*^CG^*, *ESR^AA^*/*miR-27a^AA^*, *FSHb^BB^*/*CTNNAL1^CC^*, *FSHb^BB^*/*miR-27a^AA^*, and *CTNNAL1^CG^*/*miR-27a^AB^*) were identified in another study of a Large White pig population [46]. Together, our findings provide the optimal genotype combinations between the *BMP7* and *BMP15* genes for the polygene pyramiding breeding of reproductive traits in Large White pig populations.

## 5. Conclusions

In summary, for the first time, the 3’-*UTR* of the porcine *BMP7* and *BMP15* genes was isolated and characterized. Furthermore, two point mutations (c.1569A>G in *BMP7* and c.2366G>A in *BMP15*) were identified, where c.1569A>G was found to be significantly effective on some of the sow reproductive traits in a Large White pig population. For the *BMP7* and *BMP15* genes, the *AA*/*GA* combined genotype could be the ideal model for further breeding selection. Our findings not only confirm that *BMP7* and *BMP15* are candidate genes for porcine reproductive capacity, but that they are also potentially novel genetic markers for marker-assisted selection and genome selection in pig breeding.

## Figures and Tables

**Table 1:** Primers used in this study.

	Gene	Primer Sequences (5’-3’)	Annealing Temp (°C)	Product Size (bp)	Accession No.
P1	*BMP7*	F: GTGTTCCAGGTCCACTTCAT R: CCCAACTCCAAGCAGAAA	54 °C	822	XM_005673044.3
P2	*BMP15*	F: GTGCCTATTAGCATCCTCC R: CTAACTGAAGCCTCTACCC	54 °C	1063	NM_001005155.2
P3	*BMP15*	F: TTTGAGGGAAACAGAAGG R: GTGGCTAAAGGGAACAAA	54 °C	723	NM_001005155.2

**Table 2:** Effect of the c.1569A>G polymorphism within the 3’-untranslated region of *BMP7* on the reproductive traits of the Large White pig population.

Genotypes (*n*)	Traits (LSM ± SE)			
	TNB	NBA	NSB	LW
AA (83)	12.26(±0.53) ^a^	12.07(±0.52) ^a^	0.18(±0.15) ^a^	17.00(±0.70) ^b^
AG (99)	12.10(±0.54) ^a^	11.87(±0.54) ^a^	0.23(±0.16) ^a^	18.03(±0.72) ^a^
GG (45)	11.65(±0.63) ^a^	11.60(±0.62) ^a^	0.16(±0.17) ^a^	17.40(±0.82) ^ab^

Data represent the least squares means ± SE. *n* = 227. TNB = the total number of piglets born; NBA = the total number of piglets born alive; NSB = number of stillborn; LW = litter weight. Values in each line with different lowercase superscripts are at *p* < 0.05; those with same lowercase superscripts mean that there were no differences (*p* > 0.05).

**Table 3:** Effect of the c.2366 G>A polymorphism on reproductive traits of a Large White pig population.

Genotypes (*n*)	Traits (LSM ± SE)			
	TNB	NBA	NSB	LW
GA(56)	12.56(±0.44) ^a^	12.24(±0.42) ^a^	0.21(±0.15) ^a^	17.20(±0.72) ^a^
GG(171)	12.63(±0.43) ^a^	12.34(±0.42) ^a^	0.20(±0.15) ^a^	17.57(±0.71) ^a^

Data represent the least squares means ± SE. *n* = 227. TNB = the total number of piglets born; NBA = the total number of piglets born alive; NSB = number of stillborn; LW = litter weight. No markers means that there were no differences (*p* > 0.05).

**Table 4:** Combination effect of the genotypes formed by *BMP7* c.1569A>G and *BMP15* c.2366G>A on reproductive traits.

Genotypes (*n*)	Traits (LSM ± SE)			
	TNB	NBA	NSB	LW
AA/GA(25)	12.44(±0.60) ^a^	12.21(±0.59) ^a^	0.03(±0.20) ^a^	17.08(±0.95) ^a^
AA/GG(58)	12.02(±0.59) ^ab^	11.91(±0.58) ^ab^	0.07(±0.20) ^a^	17.06(±0.93) ^a^
AG/GA(20)	11.17(±0.67) ^b^	11.04(±0.66) ^b^	0.04(±0.22) ^a^	17.04(±1.09) ^a^
AG/GG(79)	12.31(±0.56) ^a^	12.08(±0.56) ^a^	0.14(±0.19) ^a^	18.25(±0.90) ^a^
GG/GA(11)	11.81(±0.80) ^ab^	11.78(±0.80) ^ab^	0.01(±0.26) ^a^	17.36(±1.32) ^a^
GG/GG(34)	11.49(±0.69) ^ab^	11.48(±0.68) ^ab^	0.04(±0.23) ^a^	16.97(±1.09) ^a^

Data represent the least squares means ± SE. *n* = 227. TNB = the total number of piglets born; NBA = the total number of piglets born alive; NSB = number of stillborn; LW = litter weight. Values in each line with different lowercase superscripts are at *p* < 0.05. Same lowercase superscripts means that there were no differences (*p* > 0.05).

## Supplementary Materials

The following are available online at https://www.mdpi.com/2076-2615/9/11/905/s1, Supplementary Table S1: Statistical description of reproductive traits, Supplementary Table S2: Additive and dominant effect of BMP7 on the reproductive traits in a Large White pig population.

## References

[1] Ongaro L., Schang G., Ho C.C., Zhou X., Bernard D.J. (2019). TGF-beta superfamily regulation of follicle-stimulating hormone synthesis by gonadotrope cells: Is there a role for bone morphogenetic proteins?. Endocrinology.

[2] Abdurahman A., Du X., Yao Y., Sulaiman Y., Aniwashi J., Li Q. (2019). Smad4 feedback enhances BMPR1B transcription in ovine granulosa cells. Int. J. Mol. Sci..

[3] Richani D., Gilchrist R.B. (2018). The epidermal growth factor network: Role in oocyte growth, maturation and developmental competence. Hum. Reprod. Update.

[4] Wang Y., Fortin J., Lamba P., Bonomi M., Persani L., Roberson M.S., Bernard D.J. (2008). Activator protein-1 and smad proteins synergistically regulate human follicle-stimulating hormone beta-promoter activity. Endocrinology.

[5] Du X., Zhang L., Li X., Pan Z., Liu H., Li Q. (2016). TGF-beta signaling controls FSHR signaling-reduced ovarian granulosa cell apoptosis through the SMAD4/miR-143 axis. Cell Death Dis..

[6] Li Q., Du X., Pan Z., Zhang L., Li Q. (2018). The transcription factor SMAD4 and miR-10b contribute to E2 release and cell apoptosis in ovarian granulosa cells by targeting CYP19A1. Mol. Cell Endocrinol..

[7] Yan C., Wang P., DeMayo J., DeMayo F.J., Elvin J.A., Carino C., Prasad S.V., Skinner S.S., Dunbar B.S., Dube J.L. (2001). Synergistic roles of bone morphogenetic protein 15 and growth differentiation factor 9 in ovarian function. Mol. Endocrinol..

[8] Sanfins A., Rodrigues P., Albertini D.F. (2018). GDF-9 and BMP-15 direct the follicle symphony. J. Assist. Reprod. Genet..

[9] Galloway S.M., McNatty K.P., Cambridge L.M., Laitinen M.P., Juengel J.L., Jokiranta T.S., McLaren R.J., Luiro K., Dodds K.G., Montgomery G.W. (2000). Mutations in an oocyte-derived growth factor gene (BMP15) cause increased ovulation rate and infertility in a dosage-sensitive manner. Nat. Genet..

[10] Mulsant P., Lecerf F., Fabre S., Schibler L., Monget P., Lanneluc I., Pisselet C., Riquet J., Monniaux D., Callebaut I. (2001). Mutation in bone morphogenetic protein receptor-IB is associated with increased ovulation rate in Booroola Merino ewes. Proc. Natl. Acad. Sci. USA.

[11] Abdoli R., Zamani P., Mirhoseini S.Z., Ghavi H.N., Nadri S. (2016). A review on prolificacy genes in sheep. Reprod. Domest. Anim..

[12] Li W.T., Zhang M.M., Li Q.G., Tang H., Zhang L.F., Wang K.J., Zhu M.Z., Lu Y.F., Bao H.G., Zhang Y.M. (2017). Whole-genome resequencing reveals candidate mutations for pig prolificacy. Proc. Biol. Sci..

[13] Li X., Ye J., Han X., Qiao R., Li X., Lv G., Wang K. (2019). Whole-genome sequencing identifies potential candidate genes for reproductive traits in pigs. Genomics.

[14] Mayr C. (2017). Regulation by 3′-Untranslated Regions. Annu. Rev. Genet..

[15] Vejnar C.E., Abdel M.M., Takacs C.M., Yartseva V., Oikonomou P., Christiano R., Stoeckius M., Lau S., Lee M.T., Beaudoin J.D. (2019). Genome wide analysis of 3’ UTR sequence elements and proteins regulating mRNA stability during maternal-to-zygotic transition in zebrafish. Genome Res..

[16] Bartel D.P. (2018). Metazoan MicroRNAs. Cell.

[17] Yang L., Du X., Liu L., Cao Q., Pan Z., Li Q. (2019). miR-1306 Mediates the Feedback Regulation of the TGF-beta/SMAD Signaling Pathway in Granulosa Cells. Cells.

[18] Woo I., Christenson L.K., Gunewardena S., Ingles S.A., Thomas S., Ahmady A., Chung K., Bendikson K., Paulson R., McGinnis L.K. (2018). Micro-RNAs involved in cellular proliferation have altered expression profiles in granulosa of young women with diminished ovarian reserve. J. Assist. Reprod Genet..

[19] Donadeu F.X., Mohammed B.T., Ioannidis J. (2017). A miRNA target network putatively involved in follicular atresia. Domest. Anim. Endocrinol..

[20] Mao Z., Fan L., Yu Q., Luo S., Wu X., Tang J., Kang G., Tang L. (2018). Abnormality of Klotho Signaling Is Involved in Polycystic Ovary Syndrome. Reprod. Sci..

[21] Hu Z., Shen W.J., Kraemer F.B., Azhar S. (2017). Regulation of adrenal and ovarian steroidogenesis by miR-132. J. Mol. Endocrinol..

[22] Liu Z., Zhou Y., Yuan Y., Nie F., Peng R., Li Q., Lyu Z., Mao Z., Huang L., Zhou L. (2015). MiR542-3p Regulates the Epithelial-Mesenchymal Transition by Directly Targeting BMP7 in NRK52e. Int. J. Mol. Sci..

[23] Ying X., Sun Y., He P. (2017). MicroRNA-137 inhibits BMP7 to enhance the epithelial-mesenchymal transition of breast cancer cells. Oncotarget.

[24] Garcia-Maurino S.M., Rivero-Rodriguez F., Velazquez-Cruz A., Hernandez-Vellisca M., Diaz-Quintana A., De la Rosa M.A., Diaz-Moreno I. (2017). RNA Binding Protein Regulation and Cross-Talk in the Control of AU-rich mRNA Fate. Front. Mol. Biosci..

[25] Otsuka H., Fukao A., Funakami Y., Duncan K.E., Fujiwara T. (2019). Emerging Evidence of Translational Control by AU-Rich Element-Binding Proteins. Front. Genet..

[26] Abdelmohsen K., Tominaga K., Lee E.K., Srikantan S., Kang M.J., Kim M.M., Selimyan R., Martindale J.L., Yang X., Carrier F. (2011). Enhanced translation by Nucleolin via G-rich elements in coding and non-coding regions of target mRNAs. Nucleic Acids Res..

[27] Lepeta K., Purzycka K.J., Pachulska-Wieczorek K., Mitjans M., Begemann M., Vafadari B., Bijata K., Adamiak R.W., Ehrenreich H., Dziembowska M. (2017). A normal genetic variation modulates synaptic MMP-9 protein levels and the severity of schizophrenia symptoms. EMBO Mol. Med..

[28] Rajesh G., Mishra S.R., Paul A., Punetha M., Vidyalakshmi G.M., Narayanan K., Bag S., Bhure S.K., Singh C.V., Maurya V.P. (2018). Transcriptional and translational abundance of Bone morphogenetic protein (BMP) 2, 4, 6, 7 and their receptors BMPR1A, 1B and BMPR2 in buffalo ovarian follicle and the role of BMP4 and BMP7 on estrogen production and survival of cultured granulosa cells. Res. Vet. Sci..

[29] Glister C., Regan S.L., Samir M., Knight P. (2018). Gremlin, Noggin, Chordin and follistatin differentially modulate BMP induced suppression of androgen secretion by bovine ovarian theca cells. J. Mol. Endocrinol..

[30] Zhang H., Klausen C., Zhu H., Chang H.M., Leung P.C. (2015). BMP4 and BMP7 Suppress StAR and Progesterone Production via ALK3 and SMAD1/5/8-SMAD4 in Human Granulosa-Lutein Cells. Endocrinology.

[31] Rajesh G., Paul A., Mishra S.R., Bharati J., Thakur N., Mondal T., Soren S., Harikumar S., Narayanan K., Chouhan V.S. (2017). Expression and functional role of Bone Morphogenetic Proteins (BMPs) in cyclical corpus luteum in buffalo (*Bubalus bubalis*). Gen. Comp. Endocrinol..

[32] Lee W.S., Otsuka F., Moore R.K., Shimasaki S. (2001). Effect of bone morphogenetic protein-7 on folliculogenesis and ovulation in the rat. Biol. Reprod..

[33] Bahire S.V., Rajput P.K., Kumar V., Kumar D., Kataria M., Kumar S. (2019). Quantitative expression of mRNA encoding BMP/SMAD signaling genes in the ovaries of Booroola carrier and non-carrier GMM sheep. Reprod. Domest. Anim..

[34] Zhang Y., Li F., Feng X., Yang H., Zhu A., Pang J., Han L., Zhang T., Yao X., Wang F. (2017). Genome-wide analysis of DNA Methylation profiles on sheep ovaries associated with prolificacy using whole-genome Bisulfite sequencing. BMC Genom..

[35] Feng X., Xie S.Y., Zhou J.S., Sun G.R., Lu P., Li M. (2013). Polymorphisms of the bone morphogenetic protein 7 gene (BMP7) and association analysis with sow productive traits. Anim. Reprod. Sci..

[36] Christoforou E.R., Pitman J.L. (2019). Intrafollicular growth differentiation factor 9: Bone morphogenetic 15 ratio determines litter size in mammalsdagger. Biol. Reprod..

[37] Garcia P., Aspee K., Ramirez G., Dettleff P., Palomino J., Peralta O.A., Parraguez V.H., De Los R.M. (2019). Influence of growth differentiation factor 9 and bone morphogenetic protein 15 on in vitro maturation of canine oocytes. Reprod. Domest. Anim..

[38] Velasquez A., Mellisho E., Castro F.O., Rodriguez-Alvarez L. (2019). Effect of BMP15 and/or AMH during in vitro maturation of oocytes from involuntarily culled dairy cows. Mol. Reprod. Dev..

[39] Hanrahan J.P., Gregan S.M., Mulsant P., Mullen M., Davis G.H., Powell R., Galloway S.M. (2004). Mutations in the genes for oocyte-derived growth factors GDF9 and BMP15 are associated with both increased ovulation rate and sterility in Cambridge and Belclare sheep (*Ovis aries*). Biol Reprod..

[40] Roy S., Gandra D., Seger C., Biswas A., Kushnir V.A., Gleicher N., Kumar T.R., Sen A. (2018). Oocyte-Derived Factors (GDF9 and BMP15) and FSH Regulate AMH Expression Via Modulation of H3K27AC in Granulosa Cells. Endocrinology.

[41] Tang J., Hu W., Di R., Liu Q., Wang X., Zhang X., Zhang J., Chu M. (2018). Expression Analysis of the Prolific Candidate Genes, BMPR1B, BMP15, and GDF9 in Small Tail Han Ewes with Three Fecundity (FecB Gene) Genotypes. Animals.

[42] Demars J., Fabre S., Sarry J., Rossetti R., Gilbert H., Persani L., Tosser-Klopp G., Mulsant P., Nowak Z., Drobik W. (2013). Genome-wide association studies identify two novel BMP15 mutations responsible for an atypical hyperprolificacy phenotype in sheep. PLoS Genet..

[43] Lassoued N., Benkhlil Z., Woloszyn F., Rejeb A., Aouina M., Rekik M., Fabre S., Bedhiaf-Romdhani S. (2017). FecX (Bar) a Novel BMP15 mutation responsible for prolificacy and female sterility in Tunisian Barbarine Sheep. BMC Genet..

[44] Zak L.J., Gaustad A.H., Bolarin A., Broekhuijse M., Walling G.A., Knol E.F. (2017). Genetic control of complex traits, with a focus on reproduction in pigs. Mol. Reprod Dev..

[45] Panasiewicz G., Bieniek-Kobuszewska M., Lipka A., Majewska M., Jedryczko R., Szafranska B. (2017). Novel effects of identified SNPs within the porcine Pregnancy-Associated Glycoprotein gene family (pPAGs) on the major reproductive traits in Hirschmann hybrid-line sows. Res. Vet. Sci..

[46] Pang P., Li Z., Hu H., Wang L., Sun H., Mei S., Li F. (2018). Genetic effect and combined genotype effect of ESR, FSHbeta, CTNNAL1 and miR-27a loci on litter size in a Large White population. Anim. Biotechnol..

[47] Haggman J., Uimari P. (2017). Novel harmful recessive haplotypes for reproductive traits in pigs. J. Anim. Breed. Genet..

