# Supplementary material for: Variants in BMP7 and BMP15 3’-UTRs Associated with Reproductive Traits in a Large White Pig Population
Source: Animals (Basel). 2019 Nov 1;9(11):905. doi: 10.3390/ani9110905 (PMC6912256; doi:10.3390/ani9110905)
Supplement: Supplementary file 1 [file animals-09-00905-s001.pdf]

Supplementary Table legends

Supplementary Table S1 Statistical description of reproductive traits.

Supplementary Table S2 Additive and dominant effect of *BMP7* on the reproductive traits in a Large White pig population.

Supplementary Table S1

Parity	TNB				NBA				NSB				LW			
(n)	Mean	SD	Max	Min	Mean	SD	Max	Min	Mean	SD	Max	Min	Mean	SD	Max	Min
1(214)	12.16	2.55	19	6	11.93	2.54	19	6	0.15	0.45	3	0	16.66	3.70	31.3	7.6
2(208)	12.26	2.98	21	6	11.99	2.96	21	6	0.24	0.58	3	0	18.38	4.31	32.5	6.5
3(184)	13.55	2.90	21	6	12.97	2.88	19	5	0.42	1.14	8	0	19.27	4.30	33.55	7.1
4(153)	13.46	2.82	22	6	12.67	2.62	20	6	0.72	1.42	7	0	18.50	3.80	27.85	6.35
5(90)	13.50	3.27	21	6	12.69	3.14	21	5	0.71	0.68	9	0	18.57	4.84	33.2	8.2
6(39)	13.08	2.98	21	7	12.36	2.74	18	7	0.59	0.84	3	0	17.97	3.79	25	9.2
Total (227)	12.86	2.94	22	6	12.38	2.83	21	5	0.40	1.01	9	0	18.17	4.22	33.55	6.35

TNB = the total number of piglets born; NBA = he total number of piglets born alive; NSB = number of stillborn; LW = litter weight.

Supplementary Table S2

Traits	additive effect	dominant effect
TNB	-0.2021±0.2601	0.8316±0.3740*
NBA	0.0092±0.0528	0.0590±0.0755
NSB	0.2332±0.2035	0.0371±0.1489
LW	0.3048±0.2059	0.1962±0.1047

TNB = the total number of piglets born; NBA = he total number of piglets born alive; NSB = number of stillborn; LW = litter weight. Values in each box with * superscripts are at $P < 0.05$; those with no markers mean there were no differences ($P > 0.05$).
